# Imiquimod nanocrystal-loaded dissolving microneedles prepared by DLP printing

**DOI:** 10.1007/s13346-024-01567-0

**Published:** 2024-03-12

**Authors:** Eliška Petrová, Stanislav Chvíla, František Štěpánek, Jarmila Zbytovská, Dimitrios A. Lamprou

**Affiliations:** 1 School of Pharmacy, Queen’s University Belfast, 97 Lisburn Road, BT9 7BL Belfast, UK; 2 Department of Organic Technology, University of Chemistry and Technology Prague, Faculty of Chemical Technology, Technická 5, 166 28 Prague 6, Czech Republic; 3 Faculty of Chemical Technology, Department of Organic Technology, University of Chemistry and Technology Prague, Technická 5, 166 28 Prague, Czech Republic

**Keywords:** Microneedles, 3D printing, Nanocrystals, Imiquimod, Dermal delivery

## Abstract

The utilization of 3D printing– digital light processing (DLP) technique, for the direct fabrication of microneedles encounters the problem of drug solubility in printing resin, especially if it is predominantly composed of water. The possible solution how to ensure ideal belonging of drug and water-based printing resin is its pre-formulation in nanosuspension such as nanocrystals. This study investigates the feasibility of this approach on a resin containing nanocrystals of imiquimod (IMQ), an active used in (pre)cancerous skin conditions, well known for its problematic solubility and bioavailability. The resin blend of polyethylene glycol diacrylate and N-vinylpyrrolidone, and lithium phenyl-2,4,6-trimethylbenzoylphosphinate as a photoinitiator, was used, mixed with IMQ nanocrystals in water. The final microneedle-patches had 36 cylindrical microneedles arranged in a square grid, measuring approximately 600 μm in height and 500 μm in diameter. They contained 5wt% IMQ, which is equivalent to a commercially available cream. The homogeneity of IMQ distribution in the matrix was higher for nanocrystals compared to usual crystalline form. The release of IMQ from the patches was determined ex vivo in natural skin and revealed a 48% increase in efficacy for nanocrystal formulations compared to the crystalline form of IMQ.

**Graphical Abstract:**

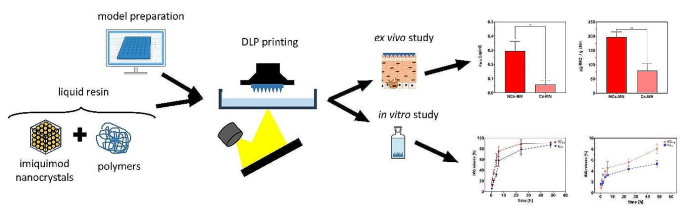

## Introduction

Microneedles (MNs) are one of the options for the enhancement of drug penetration into the skin based on a very gentle mechanical disruption of the skin barrier. MNs are very small, ranging in size from a few micrometres to a maximum of 2 mm. The application is painless because the MNs do not interact with nerve endings in the deeper layers of the skin [1, 2]. However, their size is sufficient to penetrate the *stratum corneum*, and so they can efficiently deliver drugs to deeper layers of the skin to treat skin diseases or further across the skin barrier in the case of transdermal application [3].

The standard preparation of MNs is time-consuming. The main demanding step is the creation of special moulds into which the MNs are cast. However, using 3D printing technology (3DP) can significantly reduce the time required for MNs production [4, 5]. To prepare MNs by 3DP mainly photopolymerization 3D printers such as stereolithographic (SLA) or digital light processing (DLP) are used. In both cases, light of a precise wavelength is projected into a defined space. Simultaneously, they are the most accurate printing methods [6, 7] which enable to prepare a wide range of different MN types [8, 9]. The next huge advantage of using DLP printing instead of traditional approach is the ability of change the shape and size of final MN patch very easily. This can be highly beneficial, particularly in treatment of diverse skin lesions that may vary significantly from one case to another.

For partially or fully dissolvable MNs, the drug is directly incorporated into the resin. Upon application of these MNs to the skin, the drug is released from the carrier, and simultaneously, the MNs gradually degrade, mainly through hydrolysis [10]. In most cases, the drug is dissolved in the polymer matrix, which is primary composed of water as solvent. This hydrophilic nature of the polymer matrix can be very limiting for the lipophilic molecules that are the basis of many drugs. An example of such a structure is imiquimod (IMQ).

IMQ is an immunostimulant active used in the treatment of precancerous skin conditions, particularly actinic keratosis and basal cell carcinoma. Currently, it is approved as a 5 wt% cream, but its formulation is still challenging due to its very high lipophilicity and extremely poor solubility in water and other pharmaceutical vehicles [11]. To date, research on improving the IMQ bioavailability has mostly focused on its encapsulation into various types of nanoparticles, such as microemulsions, polymeric nanoparticles [12, 13], nanosponges [14] or nanoemulsions [15]. In our recent study, we compared different types of IMQ nanocarriers. In particular, nanocrystals (NCs) were evaluated as highly effective for targeting IMQ to skin tissue [16]. Unfortunately, nanoparticulate formulations are very often liquid water dispersions which can limit their use in dermatological practice especially when precise dosing is required. However, they can contain a high IMQ content and, due to their aqueous nature, may be potentially miscible with the MN matrix used in DLP printing. This led us to the idea of combining these two techniques and incorporating IMQ NCs into the printing matrix. To our knowledge, the combination of 3D printed MNs with NCs has not yet been described for any drug.

Therefore, the aim of this research is to develop a procedure for DLP printing of MN patches loaded with IMQ in NC form and also in the usual crystalline form. These patches are characterized by different methods. In addition, their efficacy in delivering IMQ to the skin tissue is evaluated by an ex vivo permeation study. As a result, the application of two forms of IMQ, namely NC and conventional crystalline form, encapsulated in MN matrix is compared.

## Materials and methods

### Materials

Imiquimod (IMQ, ≥ 95%) was purchased from Cayman Chemical (Michigan, USA), Tween 80, vinylpyrrolidone (VP, ≥ 98%), polyethylene glycol diacrylate M_w_ = 700 g/mol (PEGDA), lithium phenyl-2,4,6-trimethylbenzoylphosphinate (LAP, ≥ 95%), methanol (≥ 99.9%), acetonitrile (≥ 99.9%), gentamicin sulphate (≥ 95%), Nile Red (≥ 97%), isopropyl alcohol (≥ 98%), ammonium acetate (≥ 98%) and phosphate-buffered saline (PBS; 0.01 M phosphate buffer, 0.0027 M potassium chloride and 0.137 M sodium chloride, pH 7.4) tablets were obtained from Merck KGaA (Darmstadt, Germany). Water was deionized, distilled, and filtered through a Millipore Q purification system.

### Preparation of nanocrystals

NCs (5 wt% in final formulation) were prepared according to Petrová etl al [16]. via small-scale wet stirred medium milling. In short, 30 mg IMQ with 7.5 mg Tween®80 and 1 ml deionized water were placed in a 25 ml amber glass vial containing the milling medium (5 g of 0.4–0.5 mm diameter zirconium oxide milling beads and a 8 × 20 mm PTFE cross stirrer). The milling was performed at room temperature at constant 700 rpm, for 24 h. After the milling period, the suspension was diluted by 1 ml of deionized water and rehomogenized at 700 rpm for 1 min in order to achieve good homogeneity of the suspension. After brief homogenization, the vial was tilted and the suspension was carefully drained using a PTFE pipette so as not to drain milling beads along with the sample. The suspension was immediately analysed via dynamic light scattering using the NANO-Flex fiber-optic instrument (Microtrac Retsch GmbH, Haan, Germany). No filtering steps were included, since satisfactory particle size distribution was obtained. The nanosuspension was subsequently freeze dried (1 h − 40 °C → 3 h − 35 °C → 5 h 35 °C, 26 Pa) using AdVantage 2.0 benchtop freeze dryer (SP Scientific, Warminster, USA). This procedure provided the freeze dried powder with residual moisture ≤ 2% and was based on the standard procedure for previously used nanoformulations [17]. The intactness of freeze-dried NC was checked by transmission electron microscopy (TEM, Jeol JEM-1010, Jeol Ltd., Tokyo, Japan).

### DLP printing and characterisation of microneedles

**Fig. 1:**
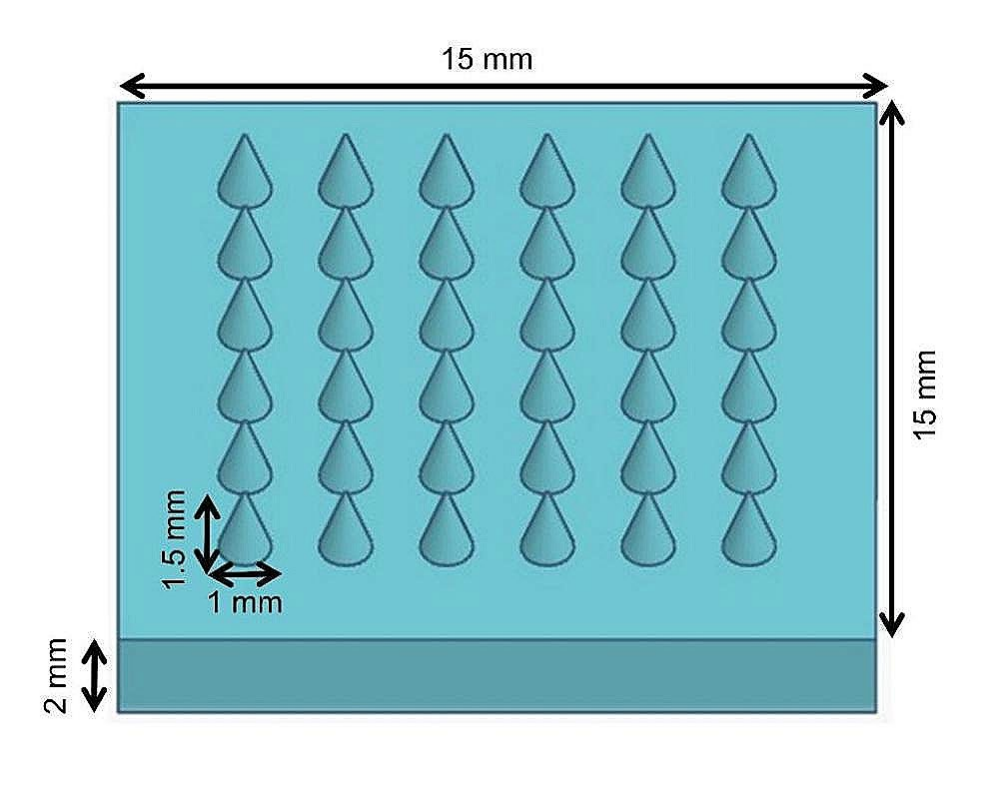
The patch CAD design (obtained by TinkerCAD software) used for the DLP printing of IMQ-loaded microneedle patches

MNs were designed by TinkerCAD (Autodesk, San Francisco, USA), an online computer-aided design software. The patch with MNs was designed as a square of 15 × 15 × 2 mm (Fig. 1). Each patch contained 36 conical MNs which were of 1 mm in diameter and 1.5 mm in height after printing (the dimensions of final patch are described in Chap. 3.1). The design was converted to.stl format and uploaded to LumenX (Cellink, Göteborg, Sweden) 3D printer. The resin used for the printing consisted of VP, PEGDA, photoiniciator– LAP (1 wt%), and distilled water as the solvent. The printing resolution was 50 μm and intensity of UV light was 22.5 mW/cm^2^ per layer.

After the printing process, the patch was gently removed, washed in 70% ethanol, and put in the UV-chamber (Asiga, Alexandria, Australia) for 1 min. Finally, the patch was dried at room temperature for 2 days, when the moisture in the patch stabilized. In the case of IMQ loaded patches, the freeze-dried IMQ NCs were added to resin and stirred for 15 min (500 rpm). The patches were visualized by optical microscopy Leica EZ4D (Leica Microsystems, Milton Keynes, UK) at room temperature.

#### Fourier-transform infrared spectroscopy (FTIR)

FTIR was used to determine the monomer conversion in MNs patches and to confirm the IMQ incorporation in the matrix and its distribution though matrix. All results were reported as average from 3 independent measurements. Spectra were recorded using a Nicolet iZ10 (Thermo Scientific, Watham, MA, USA) equipped with a single reflective Miracle ZnSe crystal (PIKE technologies, Madison, WI, USA) at constant clamping force. The spectra were collected at a resolution of 2 cm^− 1^ by co-addition of 64 scans and evaluated using the OMNIC™ software. Spectra were normalized in Origin PRO (OriginLab, Wellesley Hills, USA) to minimize the effect of variations.

#### Differential scanning calorimetry (DSC)

DSC was used to determine the thermotropic behaviour of MN patches and confirm the IMQ incorporation. All results were reported as average from 3 independent measurements.

The thermograms were measured using the Netzsch DSC 214 *Polyma* with autosampler (NETZSCH GmbH & Co.Holding KG, Selb, Germany). Samples (5–7 mg) were accurately weighed into aluminium pans and crimped with a lid. The measurements were performed under a heating range of 20 °C/min and temperature range of − 10– 400 °C. Nitrogen was used as a purge gas at flow rate 40 ml/min. As the standard, the empty aluminium pan was used. The melting points were determined as the position of the main phase transition peak in the thermogram.

#### Insertion studies in porcine skin

Fresh neonatal pig skin was kindly donated from a local slaughterhouse following all local regulations. The skin was cleaned in distilled water and stored at − 20 °C. The skin was defrosted at room temperature and placed on the microscope glass and fixed with tape on the sides. The patch was pressed in the skin with thumb pressure (~ 20 N) for 1 min. Subsequently, the patch was removed from the skin and a solution of Nile Red in isopropyl alcohol (1 wt%) was applied on the skin to visualize the holes created by MNs. The skin surface was observed by optical microscopy, with images taken before and after MNs application. The image analysis was applied for the determination of holes sized created by MNs. The experiments were performed in triplicates.

#### Insertion studies in Parafilm^®^ layers

Insertion studies in Parafilm® layers were performed according to published methods [3, 18]. 10 layers of Parafilm® were cut in the 5 × 5 cm squares and stacked on the top of each other to create a ~ 1 mm high layer as a model of the skin. A TA.XTPlus Texture Analyser (Stable Micro Systems, Surrey, UK) was used to insert the MN in the membrane by a cylindrical probe. The probe was approaching the model with the patch attached (MN pointing into the skin model) at a speed of 1.19 mm/s. Subsequently, the 32 N force was applied for 30 s. Finally, the probe automatically picked up at the same speed. Skin model with inserted MN patch was removed and the created holes were observed under optical microscope. Based on the number of created holes, the effectivity of insertion was evaluated. The study was performed in triplicates.

### Swelling study

Swelling test was conducted using the part of the patch with 6 MNs. PEGDA and VP polymerise into hydrophilic polymers. The rate of swelling is important for the IMQ release. Each patch was accurately weighted and placed in the 10 ml of buffers (PBS buffer, pH = 7.5; acetate buffer, pH = 5). At different time points (e.g., 0.5, 1, 2, 4, 6, 24 and 48 h), the patch was carefully taken off, dried with paper tissue, weighted, and placed back into the vial with buffer. The temperature of experiment was set to 32 °C. The study was performed at least in triplicates for each buffer.

### In vitro release study

For the in vitro study, only part with 6 MNs were cut out and used. The patches were placed in 15 ml vial in 15 ml of PBS buffer of pH 7.4 or in acetate buffer of pH = 5.5 (20 mM), which is the pH of the skin surface [19, 20]. The temperature was set to 32 °C (skin temperature). Samples (0.5 ml) of the dissolution media were taken at 0.5, 1, 2, 4, 6, 24 and 48 h. Then, the patch was extracted in 3 ml of methanol/acetate buffer (20 mM, pH = 4) in ratio 7:3 to determine the total residual quantity of IMQ in patch. All samples were immediately analysed by high-performance liquid chromatography (HPLC, see Chap. 2.6) and the curve of cumulative release was determined. The study was performed at least in triplicates for each buffer.

For better understanding the drug release kinetic, several mathematical models were applied [21, 22]. The coefficients were determined by Solver in Microsoft Excel.

### Ex vivo permeation studies

The study was performed in Franz diffusion cells and porcine skin was used as the tested barrier. The acceptor part was filled with PBS buffer with gentamicin (50 mg/ml, microbial protection). The skin (ca. 1 cm^2^) was placed between the donor and acceptor part. For equilibration, the cells were tempered at 32 ± 0.5 °C in a water bath for 1 h. Then, patches were pressed in the skin on the donor side and the MN patch was covered with Parafilm®. After 48 h, 300 µl of samples were taken from each cell and analysed immediately by HPLC. Then, the patch and skin were gently removed. The skin was extracted in 3 ml of the extraction medium (methanol/acetate buffer (20 mM, pH = 4) in ratio 7:3). Finally, the extracts were filtered through 0.22 μm filters and analysed by HPLC (see 2.7). Each formulation was tested in 8 skin samples.

### High-performance liquid chromatography (HPLC)

IMQ concentration was analysed by UV-HPLC Agilent Infinity 1220 LC system (Agilent Technologies Inc., Santa Clara, USA) using a flow rate of 1 ml/min, sample injection volume of 20 µl and detection wavelength of 242 nm. The mobile phase was acetonitrile/acetate buffer (pH = 4, 20 mM) in a 3:7 ratio (v/v). A Kinetex® column 150 × 4.6 mm, 5 μm, RP C18, 100 Å (Phenomenex, Torrance, USA) equipped with a guard column was used as the stationary phase. The retention time of IMQ was 3.0 min. Data were evaluated in Chemstation software 02.09 (18). A calibration curve was created from standards and the exact IMQ concentration was calculated. The method was previously validated in house for linearity, accuracy and precision.

### Statistical analysis

Statistical analysis was performed using GraphPad Prism software (GraphPad Software, Boston, USA). The Grubbs’ test was used to identify the outlier values. Unpaired t-test was used for determination of p value with confidence level 95%. The data are presented as the mean values ± SEM of minimal three independent measurements.

## Results and discussion

### DLP printing optimization

MNs were successfully prepared by DLP 3DP from the resin composed of monomers VP and PEGDA. The safety of this polymer in terms of cytotoxicity has been previously confirmed in the literature [10]. Different ratios of the monomers were tested. The development was aimed at achieving an absolute reduction in the mass of utilized polymer in the final formulation, thereby diminishing the potential presence of unreacted monomers in the final formulation. Simultaneously, the small percentage of polymers also minimized potentially possible skin irritation. Various ratios of the individual monomers VP and PEGDA were tested (e.g., 4:1, 3:1, 1:1), as well as ratios between the monomers and the solvent (distilled water). The LAP concentration remained constant.

Increasing the concentration of monomers resulted in incomplete reaction, leading to a high percentage of monomers in the resulting MN patch. Conversely, when VP content was significantly higher than PEGDA, it tended to form a hydrogel without noticeable MNs. This indicates that the ratio between VP and PEGDA is crucial for printing parameters in the presence of LAP. If there is insufficient PEGDA in the formulation, which acts as a crosslinker, MNs printing will not occur.

**Fig. 2:**
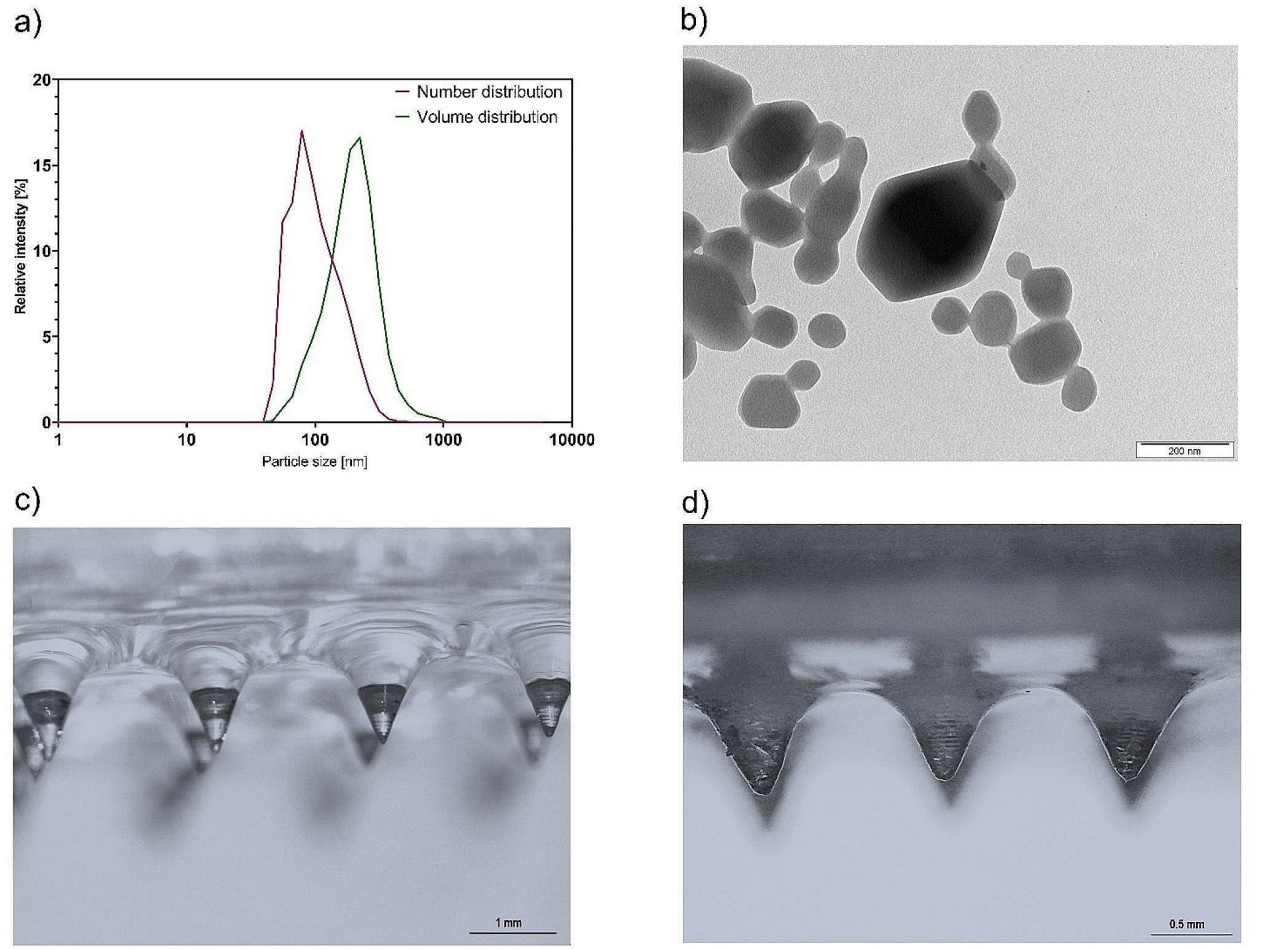
Size distribution of IMQ NCs **(A**); *n* ≥ 3, TEM images of freeze-dried IMQ NCs (**B**) and optical microscope images of the MNs immediately after printing; (**C**) and after the drying process (**D**). Magnification 8x in (**C**) and 16x in (**D**)

The final composition of printing resin for MN patch was 5 wt% PEGDA, 10 wt% VP, 1 wt% LAP and 1 wt% IMQ. The IMQ was added to the resin in the form of crystalline IMQ with size of 1.5 μm or freeze-dried NCs with size of 74 nm [16]. The stability of IMQ in presence of UV was proved and described already in literature [23]. The freeze-drying process did not influence the morphology of NCs (Fig. 2A + B).

The freshly prepared MNs were 1 330 ± 80 μm high which was visualized by optical microscope (Fig. 2C). The patch weight after printing was 531 ± 7.3 mg. After the drying process, the mean weight decreased to 93.5 ± 0.8 mg and did not change further. The final concentration of IMQ in the patch was 4.7 ± 0.5 wt% (9.4 mg cm^− 2^ patch). Based on the MN patch geometry, the combined volume of 36 MN (each MN being a cone with a base of 480 ± 30 μm in diameter and a height of 590 ± 30 μm; Fig. 2D) was approximately 3.8 mm^3^, and the volume of the rectangular base of the patch was approximately 24.4 mm^3^. Thus, out of approximately 5 mg of IMQ in a single patch, approximately 2.5% was present in the MNs and theoretically available for skin delivery after piercing through *stratum corneum*, whereas 97.5% was contained in the patch base.

#### FTIR characteristics

**Fig. 3:**
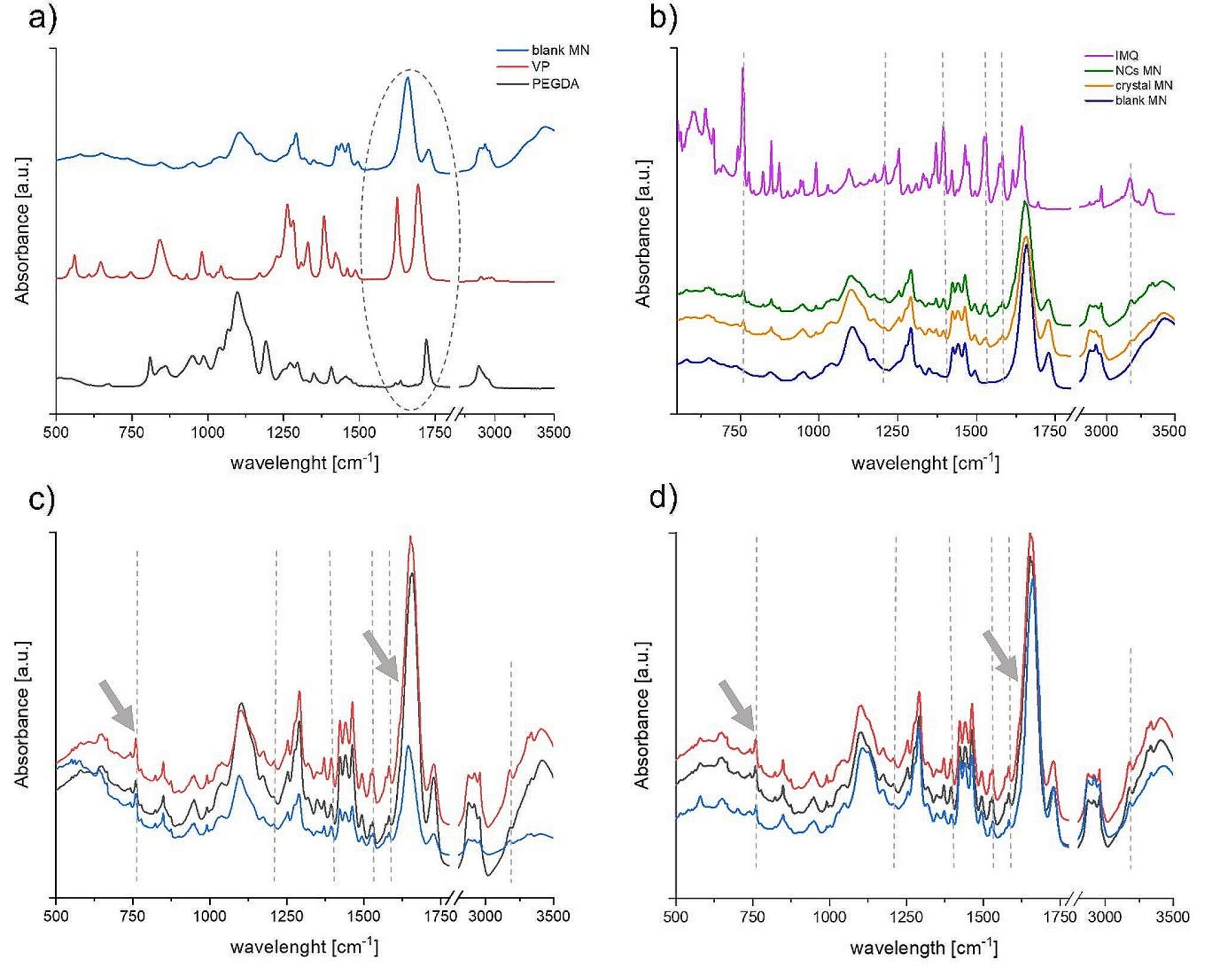
FTIR spectra of blank microneedles (MNs) and monomers (PEGDA and VP) used for the preparation with marked peaks of interest by dashed line (**A**); FTIR spectra of blank MNs, imiquimod (nano)crystals-loaded (NCs) MNs and pure imiquimod (IMQ) with marked IMQ characteristic peaks by dashed line (**B**); FTIR spectra of patches contained crystalline IMQ (**C**) and nanocrystalline IMQ (**D**) obtained in various locations of the MNs matrix. The dashed lines in C and D show peaks typical of IMQ; the arrows show the peaks of IMQ and polymer matrix used to evaluate the intensity ratio; *n* ≥ 3

FTIR was used to compare the IR signal from MN matrix with the original bulk substances. The main aim was to determine the level of monomer conversion into the polymers and possible interactions between the polymer matrix and incorporated IMQ. The IR spectra are shown in Fig. 3 and the most characteristic peaks are assigned in Table 1.

**Table 1:** Wavelength for characteristic peaks of each material in FTIR spectrum and their assignment to functional group according to literature

compound	wavelength (cm^-1^)	functional group	references
PEGDA	1097	aliphatic ether	[25]
	1720	ester	
VP	1624	alkene	[26]
	1693	ketone	[27]
patch	1105	aliphatic ether	
	1659	ketone	
	1718	ester	

Figure 3A shows the level of the polymerization process. The most intensive peak in the PEGDA spectrum can be found at 1097 and 1720 cm^-1^, which were assigned to the CO ether and CO ester stretching vibrations, respectively. VP shows a doublet at 1624 and 1693 cm^-1^ of the CC alkene and CO ketone stretching vibrations, respectively. The spectrum of the MN patch shows CO stretching of the ester and ketone groups at 1659 and 1718 cm^-1^, respectively. However, the alkene peak at 1654 cm^-1^ is missing which suggests a high polymer conversion in the matrix.

Although the VP photopolymerization is slower compared to acrylates, namely PEGDA, combination of both polymers leads to an increase in the overall conversion rate [24].

Bulk IMQ shows typical peaks at 757, 1209, 1394, 1529, 1581 and 3182 cm^− 1^ (Fig. 3B). When compared the spectra of blank MN patch and both systems with IMQ (the crystal and NC one) it can be concluded that IMQ is slightly distinct in both loaded patches. No shifts in the position of IMQ peaks were detected.

By examining spectra obtained from various patch locations or across different patches (see Fig. 3C + D), a difference between MNs containing IMQ in crystalline and NC form can be observed. The spectra of NC-MNs show a higher degree of homogeneity compared to the spectra of MNs containing crystalline IMQ. The spectra of NC-MNs from different locations are almost identical. However, when IMQ is present in the usual crystalline form, the typical peaks of IMQ (757 cm^− 1^) and the polymer matrix (1659 cm^− 1^) (see arrows in Fig. 3C) differ in their intensity ratios. This suggests that nanocrystalline IMQ is more homogeneously distributed across the patch matrix which is a condition for uniform release of IMQ.

#### DSC characteristics

**Fig. 4:**
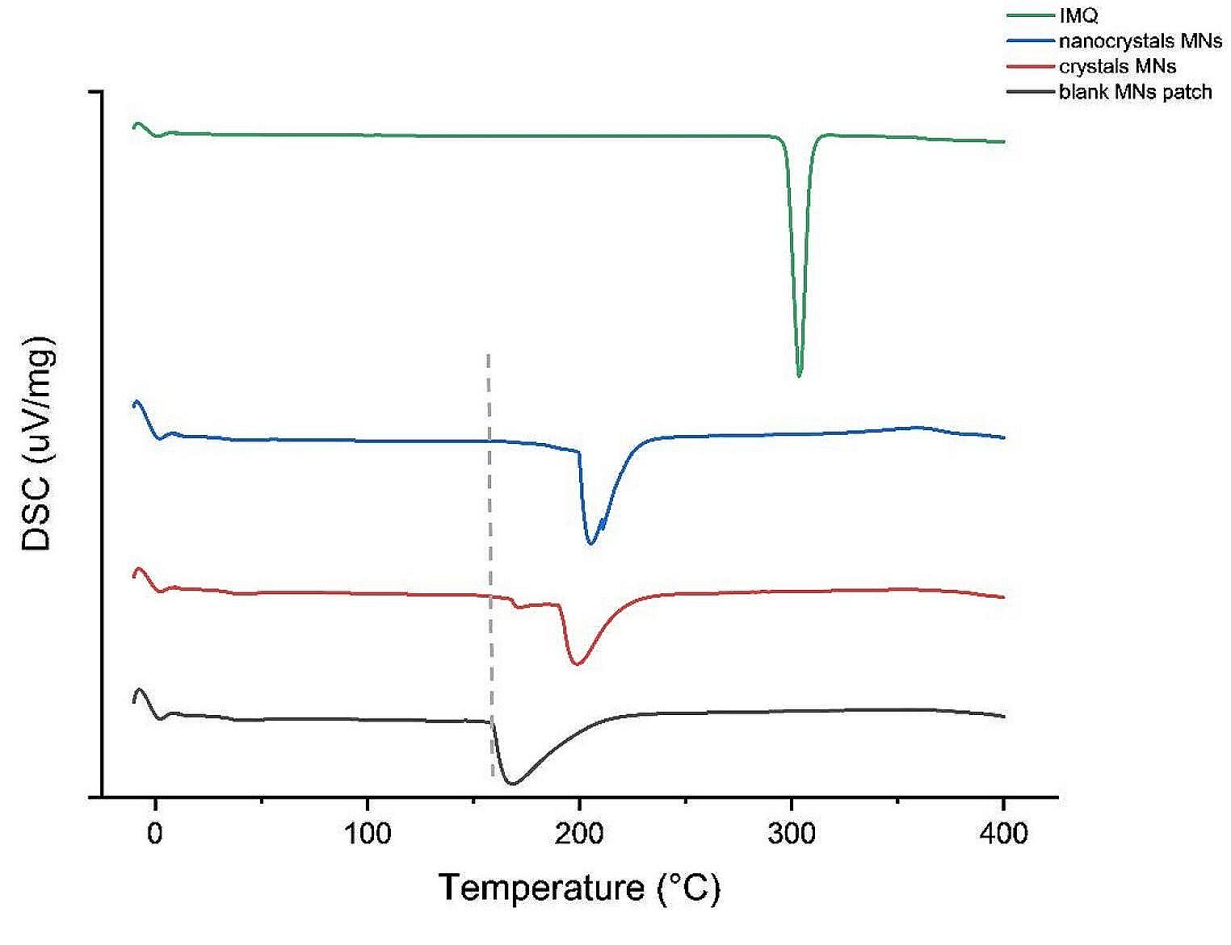
DSC curve of blank MNs, crystalline MNs and NC-MNs patches and of pure IMQ. The dashed line shows the shift in the onset of the temperature transition of the polymer matrix; *n* ≥ 3

Differential scanning calorimetry was used to determine thermotropic phase behaviour of blank MNs and to characterize the effects of incorporated IMQ in crystalline or NC form in the polymer matrix (Fig. 4). The phase transition of blank MNs was determined at 166 ± 1 °C. The melting point of pure IMQ was detected at 303 ± 1 °C. When IMQ was added to the polymer matrix, its typical melting peak vanished. Compared to the neat polymer, both IMQ systems revealed an increase in the polymer main phase transition. The crystal MNs showed the main phase transition with a small pretransition at 198 ± 1 °C and 171 ± 2 °C, respectively. The NC-MNs showed only one melting peak at 182 ± 6 °C. This indicates a good incorporation of IMQ into the polymer matrix. Compared to the crystal MNs, the absence of the pretransition in the NC-MNs hints to higher miscibility of IMQ with the surrounding polymer.

#### Insertion of MN patches

**Fig. 5:**
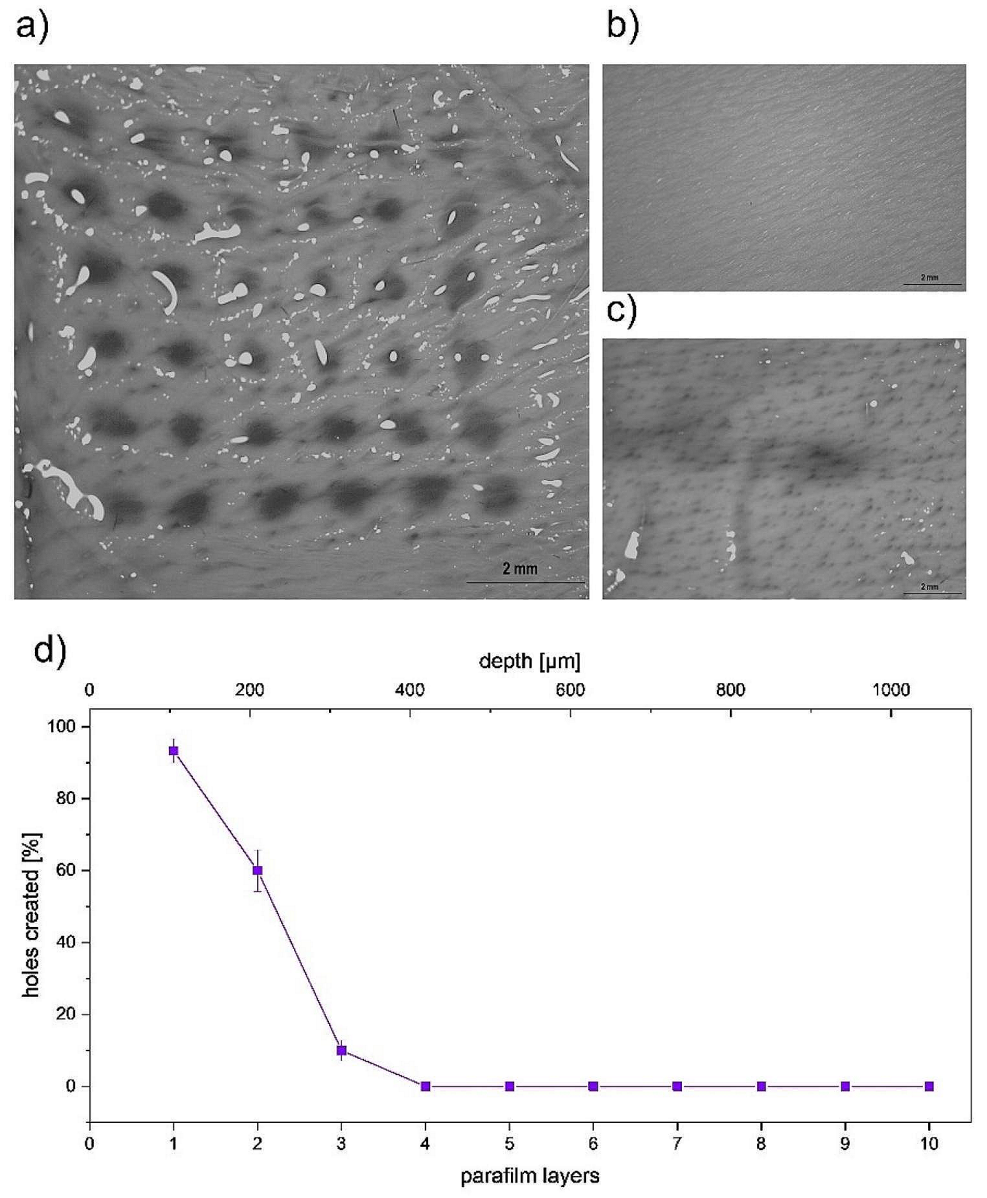
MNs penetration in porcine skin visualized by Nile Red solution after their removal (**A**), porcine skin before the MNs application (**B**), control measurement of skin not exposed to MNs (**C**), and percentage of holes created by insertion of MNs in Parafilm® layers (**D**); *n* ≥ 3

The ability of skin disruption was evaluated in porcine skin (Fig. 5B) using Nile Red for visualisation [8, 28]. MNs (Fig. 5A) breached successfully the skin and created regular holes with diameter of 0.27 ± 0.02 mm. Compared to this, control application of Nile red on intact skin showed no effect (Fig. 5C). These results indicate that the MNs can penetrate the *stratum corneum* relatively easily.

MNs insertion was also studied on the skin model based on Parafilm® (Fig. 5D). The MNs created holes in the 1st Parafilm® layer, which suggest their ability to disrupt the upper skin layer– *stratum corneum* [3]. Taken together, a maximum of 3 layers (approximately 300 μm deep in the skin) of Parafilm® were disturbed, although in the 3rd layer only around 15% of MNs penetrated (Fig. 5D). Overall, these results suggest that MNs are able to disrupt the skin barrier and thus facilitate penetration of IMQ into the deeper layers of the skin.

### Swelling studies

**Fig. 6:**
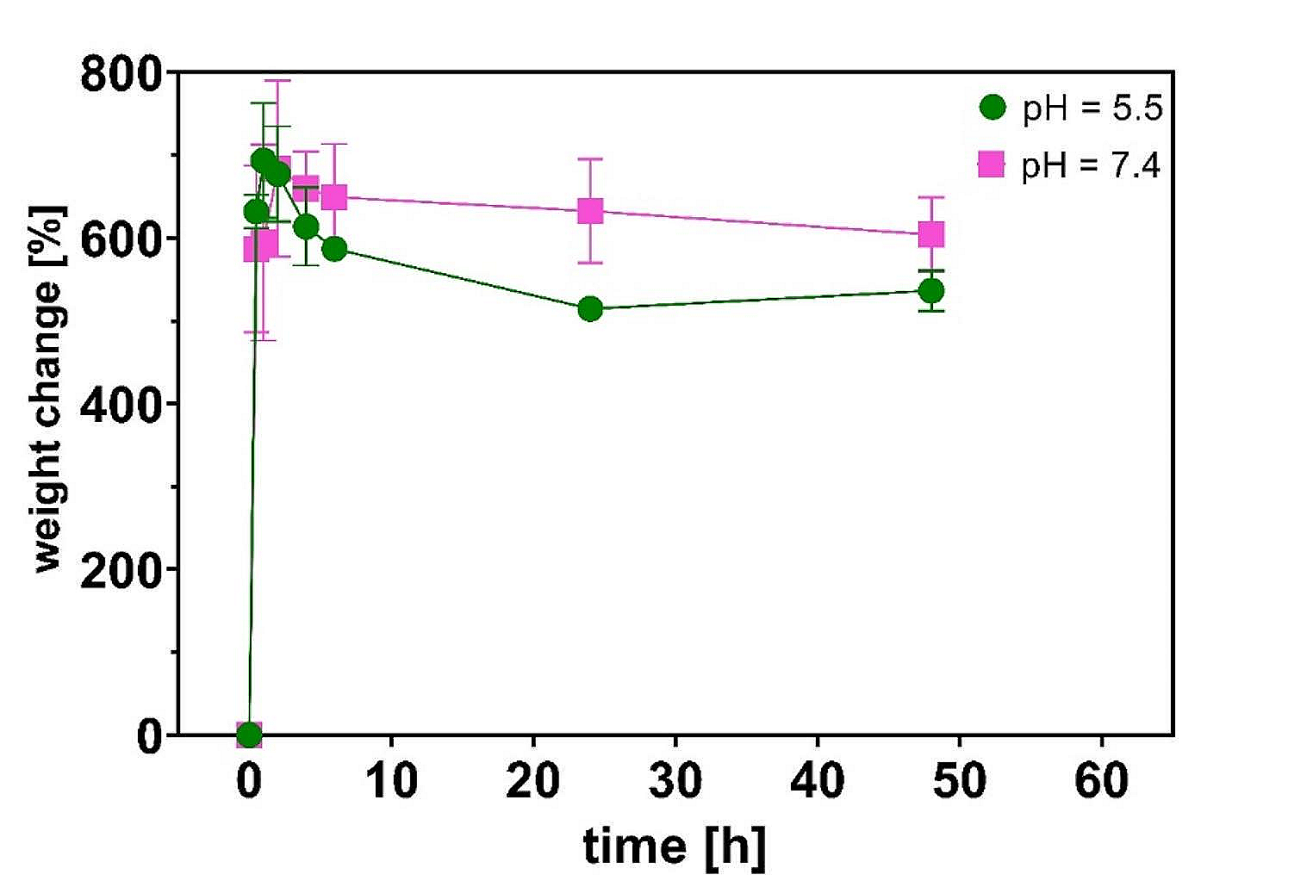
Swelling ratio of patches at pH = 5.5 (green line) and pH = 7.4 (purple line); *n* ≥ 3

Swelling of the MNs patches at pH 7.4 and 5.5 was monitored (Fig. 6). It can be observed that the most intense swelling occurred during the first hour of the experiment regardless of pH of the medium. Similar but not as intense swelling was observed by [10]. These authors report a different photoinitiator (phenylbis (2,4,6-trimethylbenzoyl) phosphine oxide) and no water in their polymer matrix which could account for the lesser swelling they observed. During the further course of our swelling experiment, the weight of the patch decreased slightly, which was more pronounced in the acidic environment. This phenomenon was probably caused by a very slow hydrolysis of the polymer to polyethylene glycol and poly(acrylate-co-vinylpyrrolidone) monomers [29].

### In vitro release

**Fig. 7:**
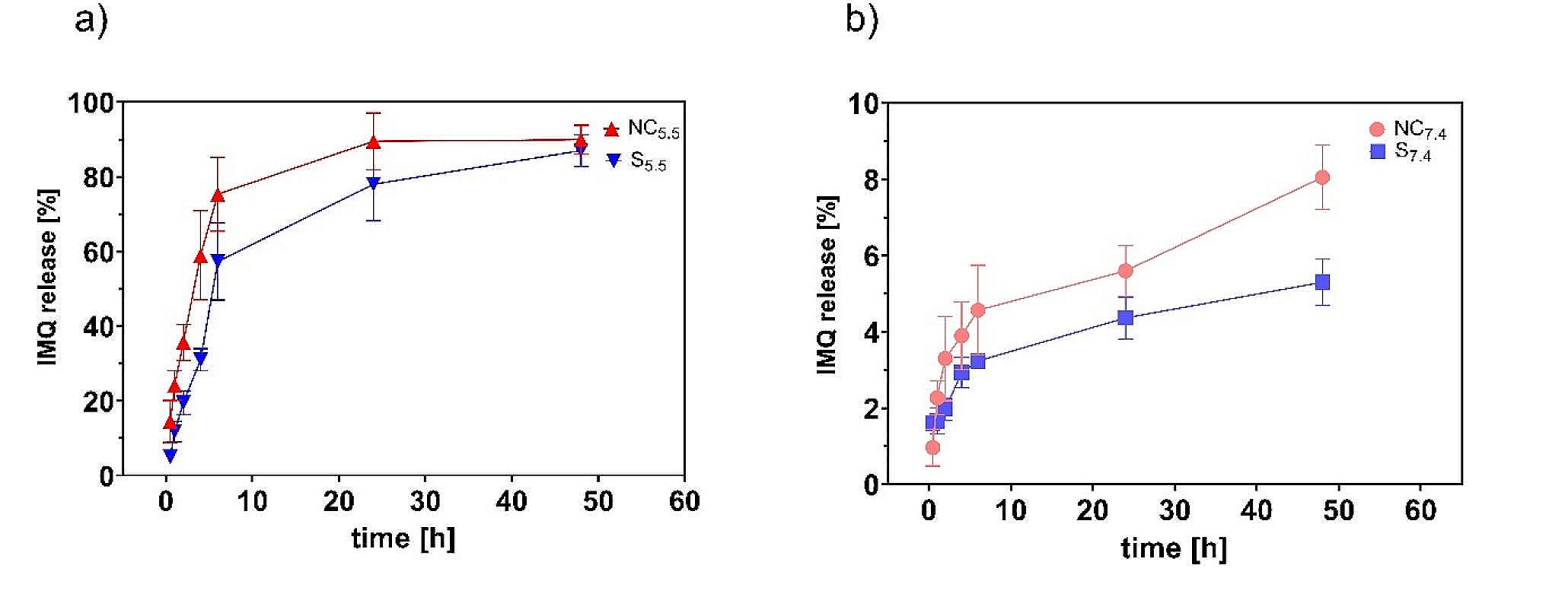
In vitro release of IMQ from the MN patches at pH = 5.5 (**A**) and pH = 7.4 (**B**); the red line belong to the NC-MNs and blue line to crystalline MNs (S); *n* ≥ 3

To evaluate the IMQ release from MNs patches, two different pH values of the dissolution media were used, namely pH 7.4 imitating the pH of the intrinsic environment and 5.5 imitating the skin surface. The release at pH 7.4 was very low compared to 5.5 (Fig. 7) and reached only 8% in 48 h (for the NC-MNs). This corresponds to the much lower solubility of IMQ at neutral or higher pH values [16]. The higher solubility of IMQ in lower pH (5.5 in our case) is caused by protonation of amine (pKa_IMQ_ = 7.3) [30]. This is responsible for the observed difference in the release of IMQ at these two pHs.

This findings also indicates that IMQ permeation though skin into the blood stream can be expected to be very low. At the same time, it is still possible to see the difference between the NC and crystal MNs. Compared to the 8% for NC, the crystalline formulation reached only around 5% (Fig. 7B).

Higher IMQ solubility at lower pH values is reflected in the dissolution profiles measured at pH 5.5. For both types of patches, around 90% of the incorporated IMQ was released in 48 h. Also at this pH, a significant difference between the NC and crystalline formulation can be found. NC-MNs released IMQ faster, reaching the maximum already at 24 h.

Both dissolution profiles at pH 5.5 show a burst effect in the first six hours of the experiment when the IMQ release reaches 60% and 75% for the crystalline and NC formulations. This trend corresponds to data published earlier [10]. In this case, the highest amount (~ 75%) of small peptide incorporated in MNs was also released during the first 6 h of the experiment. In contrast to our patches, the small peptide was fully dissolved in the polymer matrix. This indicates that MN containing NC can attain comparable effects as for dissolved drugs.

The IMQ release profile from NC-MNs obtained at pH 5.5 was approximated by various functions. The best-fitting mathematical model was determined based on the value of the coefficient of determination, calculated by minimizing the root mean square error. Table 2 shows that the most suitable models were the First Order and Hopfenberg functions. The Hopfenberg model describes the release from polymers which degrade or erode during the drug release [31–33]. This implies that the limiting factors of the IMQ release are the erosion of the matrix and the time derived from internal and external diffusion resistance related to the concentration gradient. This fact is also supported by the good fit with the first-order kinetics, which is primarily dependent on the concentration gradient.

**Table 2:** List of used mathematical models for the modelling of in vitro release in acetate buffer (pH = 5.5) with calculated coefficients of determination by Solver in Excel, the most appropriate model is in bold

Mathematic model	R^2^
**First Order**	**0.997**
Second Order	0.987
Higuchi	0.870
Hixon-Cronwell	0.530
Weibull	0.991
Gompretz	0.989
**Hopfenberg**	**0.997**

### Ex vivo permeation study

**Fig. 8:**
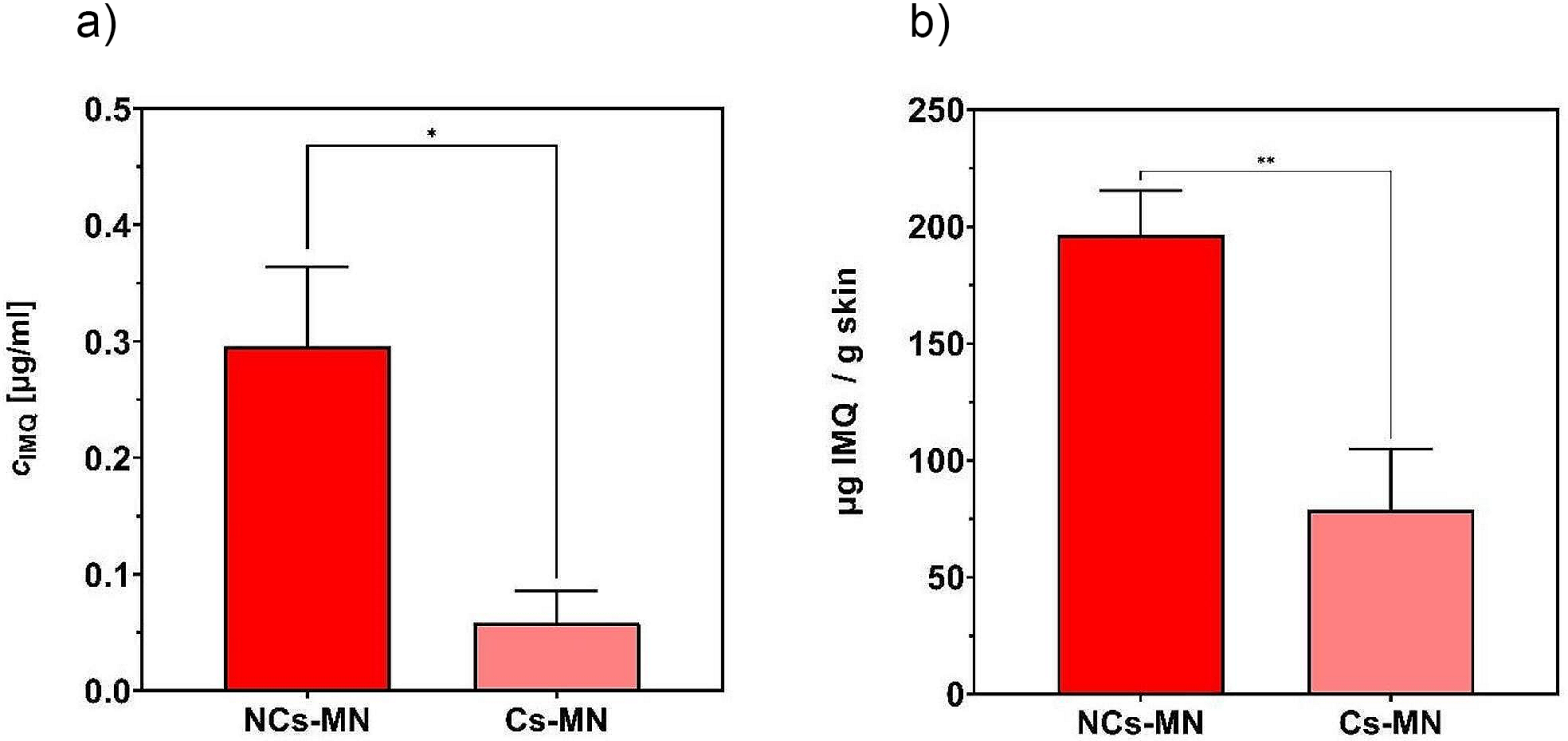
IMQ concentration in the acceptor phase (**A**); IMQ entrapment in the skin (**B**); *n* = 8; the number of asterisks show the level of significance for comparison of MNs patches with each other (p < *0.0332, **0.0021)

To assess the efficiency of the developed MN to deliver IMQ into and through the skin, the ex vivo permeation study was performed. Two parameters were evaluated, namely the concentration of IMQ in the skin tissue to estimate the dermal accumulation and the concentration of IMQ in the acceptor phase characterizing the transdermal permeation (Fig. 8). Both types of MN patches were able to deliver IMQ to the skin and also to the acceptor compartment, with NC-MNs demonstrating their superiority compared to their crystalline counterparts. The IMQ amount in the skin tissue reached 196.6 ± 18.9 and 79.1 ± 25.9 µg g^− 1^ skin for the NC and crystal MN, respectively (Fig. 8B). The final concentration in the acceptor phase was 0.30 ± 0.07 and 0.06 ± 0.03 µg ml^− 1^ for the NC and crystal MNs, respectively (Fig. 8A). NC-MNs delivered 2.5 times more IMQ in the skin compared to the control crystalline MN. This is probably thanks to the better distribution of NC in the patch (see Chap. 3.1.1) compared to the patch with crystalline IMQ. The NC also have much larger surface area, which causes faster and more efficient dissolution of IMQ in the patch and thus faster absorption in the skin.

Although this basic research is far from the development of the final product, we were interested to see to what extent our data were comparable to other developed or marketed formulations. In a recent study nanoparticulate formulations based on various nanoparticles and nanocrystals are presented and their effectivity is compared with Aldara®, the marketed cream [16]. Using analogous experimental design as in our study, Aldara® reached 34 ± 5 µg of IMQ per g of the skin tissue and 1.9 ± 0.2 µg ml^− 1^ in the acceptor phase. In this comparison, both our MN patches can be considered as very successful. Especially, the NC MN demonstrated more than five times the concentration of IMQ in the skin and six times lower IMQ concentration in the acceptor phase compared to Aldara®. Another study focused on fabrication of MN which contained IMQ in dissolved form [34]. These MN showed similar efficiency to deliver IMQ into the skin tissue as for Aldara®. One of the reasons can be much lower IMQ concentration reached in the polymer matrix of the MN than in our formulations. This clearly underlines the great potential of NC, and particularly NC formulated into MN.

The developed MN with nanocrystalline IMQ can provide efficient targeting of drugs into the dermal region with simultaneous minimizing the systemic absorption. Such MN offer not only high efficacy, but also other positive aspects such as ease of application of patches compared to liquid or semi-solid formulations and minimizing the toxicity risks of the highly potent drug.

## Conclusions

This study demonstrates the successful integration of NC into the DLP-printed MN patches. Overall, the pre-formulation of IMQ in nanocrystals and their very good compatibility with printing resin showed the way for drugs which are insoluble in the printing resins, but simultaneously they are candidates for topical application via MN. The presence of NC in the resin did not influence the printing parameters significantly. The ability to create model patches featuring 36 MN, each with a height of around 600 μm, underlined the competence of the manufacturing process.

Thorough characterization of the patches by DSC and FTIR affirmed the complete uniform integration of IMQ NC in the polymer matrix. In vitro insertion tests showed the ability of the prepared MN to sufficiently penetrate the skin barrier.

Subsequent in vitro release study revealed higher dissolution rate from NC MN compared to the crystalline formulation. The profile showed a burst effect within the first 6 h. After comparing our NC release profiles with those found in literature [10], which include substances fully dissolved in MN patches, we can conclude that the complete dissolution of the active in the printing matrix is not a limiting factor for utilizing it in DLP printing. Compared to the crystalline form, the NC-MNs also showed higher efficiency in the ex vivo permeation study to target IMQ into the skin tissue. Comparative analysis of the ex vivo study results with published data on commercially available cream confirmed that the MN patches with IMQ NC were approximately five times more effective [16]. Despite lower transdermal skin penetration, a significant increase in targeting into the skin tissue was evident.

In summary, this study highlights the potential of using NC in 3D printed MN as the innovative solution for drugs insoluble in DLP printing matrix. In particular, this approach holds significant promise for targeted applications, especially in cases such as basal cell carcinoma. Given the varying sizes of carcinomas in individual patients, the utilization of precisely tailored patches, crafted with advanced 3D technology, becomes particularly advantageous. In conjunction with appropriate polymers and controlled release mechanisms, this innovation could significantly minimize patient visits to hospitals and greatly reduce direct exposure to potent agents such as IMQ and associated risks.

## Data Availability

The datasets generated during and/or analysed during the current study are available from the corresponding author on reasonable request.

## Abbreviations

DLP: digital light processing
DSC: differential scanning microscopy
FTIR: Fourier transform infrared spectroscopy
HPLC: high-performance liquid chromatography
IMQ: imiquimod
LAP: lithium phenyl-2,4,6-trimethylbenzoylphosphinate
NC: nanocrystal
MN: microneedle
NC-MNs: nanocrystal loaded microneedles patch
PEGDA: polyethylene glycol diacrylate
PBS: phosphate-buffered saline
SLA: stereolithography
TEM: transmission electron microscopy
VP: vinylpyrrolidone
